# Illumina Next Generation Sequencing for the Analysis of *Eimeria* Populations in Commercial Broilers and Indigenous Chickens

**DOI:** 10.3389/fvets.2018.00176

**Published:** 2018-07-30

**Authors:** Ankit T. Hinsu, Jalpa R. Thakkar, Prakash G. Koringa, Vladimir Vrba, Subhash J. Jakhesara, Androniki Psifidi, Javier Guitian, Fiona M. Tomley, Dharamsibhai N. Rank, Muthusamy Raman, Chaitanya G. Joshi, Damer P. Blake

**Affiliations:** ^1^Department of Animal Genetics and Breeding, College of Veterinary Science and Animal Husbandry, Anand Agricultural University, Anand, India; ^2^Department of Animal Biotechnology, College of Veterinary Science and Animal Husbandry, Anand Agricultural University, Anand, India; ^3^Eimeria Pty Ltd, Faculty of Veterinary and Agricultural Sciences, University of Melbourne, Werribee, VIC, Australia; ^4^Department of Clinical Science and Services, Royal Veterinary College, North Mymms, Hertfordshire, United Kingdom; ^5^The Roslin Institute, University of Edinburgh, Easter Bush, Midlothian, United Kingdom; ^6^Department of Pathobiology and Population Sciences, Royal Veterinary College, North Mymms, Hertfordshire, United Kingdom; ^7^Department of Veterinary Parasitology, Madras Veterinary College, Tamil Nadu Veterinary and Animal Sciences University, Chennai, India; ^8^Translational Research Platform for Veterinary Biologicals, Tamil Nadu Veterinary and Animal Sciences University, Chennai, India

**Keywords:** next generation sequencing, chickens, *Eimeria*, 18S rRNA gene, India

## Abstract

*Eimeria* species parasites can cause the enteric disease coccidiosis, most notably in chickens where the economic and welfare implications are significant. Seven *Eimeria* species are recognized to infect chickens, although understanding of their regional occurrence, abundance, and population structure remains limited. Reports of *Eimeria* circulating in chickens across much of the southern hemisphere with cryptic genotypes and the capacity to escape current anticoccidial vaccines have revealed unexpected levels of complexity. Consequently, it is important to supplement validated species-specific molecular diagnostics with new genus-level tools. Here, we report the application of Illumina MiSeq deep sequencing to partial 18S rDNA amplicons generated using *Eimeria* genus-specific primers from chicken caecal contents collected in India. Commercial Cobb400 broiler and indigenous Kadaknath type chickens were sampled under field conditions after co-rearing (mixed type farms, *n* = 150 chickens for each) or separate rearing (single type farms, *n* = 150 each). Comparison of MiSeq results with established Internal Transcribed Spacer (ITS) and Sequence Characterised Amplified Region (SCAR) quantitative PCR assays suggest greater sensitivity for the MiSeq approach. The caecal-dwelling *Eimeria tenella* and *E. necatrix* dominated each sample set, although all seven species which infect chickens were detected. Two of the three cryptic *Eimeria* genotypes were detected including OTU-X and OTU-Y, the most northern report for the latter to date. Low levels of DNA representing other *Eimeria* species were detected, possibly representing farm-level contamination with non-replicating oocysts or *Eimeria* DNA, or false positives, indicating a requirement for additional validation. Next generation deep amplicon sequencing offers a valuable resource for future *Eimeria* studies.

## Introduction

Protozoan parasites of the genus *Eimeria* can cause the enteric disease coccidiosis. All livestock are susceptible to specific *Eimeria* species, and those which infect chickens have the greatest economic impact (1). Intensification of poultry production has elevated the significance of coccidiosis because high-density rearing of susceptible chickens increases parasite transmission, environmental contamination and risk of infection (2). Control of coccidiosis requires good husbandry as well as routine chemoprophylaxis and/or live parasite vaccination. However, *Eimeria* parasites remain widespread and drug resistance is common (3, 4), resulting in very high levels of sub-clinical infection as well as outbreaks of clinical disease. Seven *Eimeria* species have long been recognized to infect chickens; but the recent detection of *Eimeria* circulating in chickens across much of the southern hemisphere with cryptic genotypes and the capacity to escape current anticoccidial vaccines has revealed unexpected levels of complexity (3, 5, 6). As a consequence understanding *Eimeria* occurrence, abundance and population structure is increasingly important (7). Traditionally, identification of each *Eimeria* species has been achieved through a combination of oocyst morphology and/or pathological assessment of the infected intestine (8). However, these approaches require specialist expertise and can be time consuming and subjective. Molecular assays based on polymerase chain reaction (PCR), quantitative PCR (qPCR) and loop-mediated isothermal amplification (LAMP) (9–12) can overcome these problems, but are limited by variation in target sequence diversity and laborious template preparation.

Next-generation sequencing (NGS) technologies provide powerful tools for the characterization and quantification of microbial communities, and deep sequencing of bacterial 16S rDNA amplicons (microbiome sequencing) is well established (13–15). However, the transition of such approaches to eukaryotic parasites has been slow. NGS deep sequencing of internal transcribed spacer (ITS)-2 or 18S rDNA amplicons has been used to define nematode populations and detect *Toxoplasma gondii*, and was recently applied to *Eimeria* communities sampled from wildlife (16–18), but is yet to be established for *Eimeria* which infect chickens. A range of genomic targets including 18S rDNA, ITS-1 and−2, and mitochondrial cytochrome c oxidase subunit I (mtCOI) have been exploited for use as molecular diagnostics for various *Eimeria* species (19, 20) and all may be appropriate for NGS. While it has been suggested that mtCOI is the best phylogenetic marker, 18S rDNA is currently the most commonly sequenced *Eimeria* gene with the largest public dataset of reference sequences (21, 22). The work in the present study has built on these resources and describes the validation of an assay targeting 18S rDNA for NGS (Illumina MiSeq) analysis of *Eimeria* populations from chickens, comparing the assay with two well-established PCR-based protocols. Application of the NGS technique to DNA samples collected previously for a large cohort bacterial microbiome field study is used to explore the occurrence and abundance of *Eimeria* species parasites (and other closely related organisms) in caecal contents from commercial Cobb400 broilers and indigenous Kadaknath chickens reared under commercial conditions in west India.

## Materials and methods

### Ethical statement

This study was carried out using welfare standards consistent with those established under the Animals (Scientific Procedures) Act 1986, an Act of Parliament of the United Kingdom. All protocols were approved by the Ethical Review Panel of Anand Agricultural University (AAU) and the Clinical Research Ethical Review Board (CRERB) of the Royal Veterinary College. Participating farmers were informed of the objectives of the study and written consent was obtained for the same.

### Animals

Two different types of chicken were selected for use in this work. The first, the Cobb400, is a commercial broiler line used widely in India that is descended from Cobb500 and Cobb100 hybrids, the latter of which had previously been acclimatized within India (23). The second was the indigenous Indian Kadaknath chicken, a breed prized for its “black” meat, reported to be resistant to some infectious diseases (24, 25), and to present immune parameters distinct from those of modern commercial broiler chickens (26, 27).

### Experimental design

Two experimental sets were sampled in this study. In the first set, Cobb400 and Kadaknath chickens were reared together on small-scale or medium-sized commercial farms (referred to as “mixed farms,” representing flock sizes between 11 and 4,000 chickens; Supplementary Table 1), allowing direct comparisons to be made between them. Thirty farms were recruited to this set, and ten 1-day old chicks of each type were supplied to each farm and reared together using prevailing local husbandry protocols. Subsequently, five chickens of each type were culled and sampled between 36 and 40 days of age from each farm, resulting in a first set of 300 samples. Cobb400 and Kadaknath chickens sampled in this experimental set were coded CK_C and CK_K, indicating that these were from the mixed farm Cobb400/Kadaknath set, of the Cobb400 line or Kadaknath breed, respectively. In the second experimental set, chickens were sampled directly from small-scale or medium-sized commercial farms that specialised in either Cobb400 (coded C) or Kadaknath (coded K) production (referred to as “single chicken type farms,” flock sizes between 16 and 16,230 chickens) in order to increase the sample size. Again, 30 farms were recruited (15 C and 15 K) and ten birds were culled and sampled from each between 35 and 45 days of age, resulting in a second set of 300 samples. All farms were identified and recruited opportunistically and none used anticoccidial vaccines. Anticoccidial drugs were used on several farms, but the study was designed to provide a snapshot of parasite occurrence under existing husbandry systems, not the efficacy of anticoccidial prophylaxis. The presence of coccidial lesions was not scored. The samples used here were collected for use in a separate caecal microbiome study (manuscript under review). Complementary samples from other intestinal locations and faecal material from each sampled chicken were not available.

### Sample collection and DNA extraction

Birds were culled by cervical dislocation, caecal pouches were removed immediately post-mortem using sterile scissors and the caecal contents recovered by squeezing into sterile cryovials (one per chicken) containing Bacterial Protect RNA reagent (Qiagen, Germany) at an approximate ratio of 1:1. Each sample was stored and transported to the laboratory in a portable freezer at −20°C and then stored at −80°C prior to further processing. Total genomic DNA (gDNA) was extracted using a QIAamp Fast DNA Stool Mini kit (Qiagen, Germany) following the manufacturer's instructions with minor modifications. Briefly, 500 μL of caecal content mixed with Bacterial Protect RNA reagent was added to 1 mL InhibitEX buffer and homogenized by vortexing at maximum speed (3,000 rpm) for 5 min, then incubated at 80°C for 10 min. The mixture was centrifuged at 2,600 g for 1 min to remove residual solid material and 600 μL of supernatant was processed as recommended by the manufacturer. gDNA was treated with DNase free RNase (Macherey-Nagel, Germany) to remove contaminating RNA. gDNA concentration and quality were assessed using a Qubit 2.0 fluorometer (Invitrogen, ThermoFisher scientific, MA) and 0.8% (w/v) agarose (SeaKem® LE Agarose, Lonza, Switzerland) gel electrophoresis (in 0.5x TBE buffer) respectively. gDNA was stored at −20°C until further processing.

### 18s rDNA amplification and MiSeq sequencing

For 18S rDNA amplification, a pan-*Eimeria* genus specific primer pair (Forward: 5′-CGCGCAAATTACCCAATGAA-3′ and Reverse: 5′-ATGCCCCCAACTGTCCCTAT-3′) was designed for use in this study. Briefly, references representing 18S rRNA gene sequences from *Eimeria* species which infect chickens, turkeys, cattle and mice were downloaded from GenBank (accession numbers AF080614, EF210324-25, EF122251, HG793039-45, U67116, U67118-20, and U77084) and aligned using ClustalX with default parameters (28). Primers were designed using Primer3 (29), targeting non-polymorphic regions which flanked the region of greatest diversity, resulting in an amplicon of ~455 base pairs. Approximately 100 ng gDNA was used as template for PCR amplification. Each 25 μL PCR reaction mixture comprised of 2.5 μL gDNA (~40 ng/μL), 0.5 μL each forward and reverse primer (10 pM) and 12.5 μL 2X KAPA HiFi HotStart Ready Mix (Kapa Biosystems, UK). No template reactions were included to provide negative control. PCR amplification cycles were: initial denaturation at 95°C for 3 min, followed by 34 cycles of 98°C for 20 s, 65°C for 10 s and 72°C for 12 s, and a final extension at 72°C for 40 s. Amplicons were further processed for library preparation following the Illumina 16S metagenomic protocol using Illumina's Nextera XT index kit (Illumina, SD). 2 × 250 bp chemistry was used for sequencing on the Illumina MiSeq platform at the Niche Area of Excellence in the Plant Biotechnology Department, Anand Agricultural University, Anand, Gujarat. Two MiSeq runs were carried out, including 300 samples in each run. PhiX genomic control DNA was included in each run, with error rates of 3.5 and 3.77% for R1 and R2 in run 1, and 1.78 and 2.54% in run 2.

### 18s rDNA amplicon data analysis

Sequence reads were merged using PAired-eNDAssembler for Illumina sequences (PANDASeq) (30) and further analyzed using the Quantitative Insights Into Microbial Ecology (QIIME) (1.8) pipeline (31). The assembled sequences were clustered into operational taxonomic units (OTU) at 97% similarity using UCLUST through *pick_de_novo_otus.py* and *pick_closed_reference_otus.py* scripts. The cut-off was set at 99% based upon the lowest pairwise distance between reference 18S rDNA amplicon sequences, calculated by estimating the evolutionary divergence between sequences using MEGA7 (32). Analysis was conducted using the Maximum Composite Likelihood model with all positions containing gaps and missing data eliminated, leaving a total of 448 positions in the final dataset (Supplementary Table 2). A custom database was prepared from complete 18s rDNA sequences of organisms classified under order Eucoccidiorida available at NCBI (retrieved 30th October, 2017), supplemented with sequences representing the cryptic *Eimeria* genotypes OTUs X, Y and Z (3) (accession numbers LT964972-LT964974). Representative sequences from each cluster were assigned taxonomy with UCLUST consensus taxonomy assigner through *assign_taxonomy.py* script from the representative database. The results were used for calculating alpha and beta diversity indices using PAST (33). Alpha diversity was measured by calculating the Observed taxa, Dominance, Simpson, Shannon, Berger-Parker and Chao-1 diversity indices. The observed taxa represented the total number of species identified within sample groups. Dominance, ranging from 0 to 1, indicated the level of dominance by single taxa within each community. The Simpson index measured the evenness of the community, while Shannon showed entropy and considered both the number of individuals as well as the number of taxa. The Berger-Parker showed dominance by the number of individuals in the dominant taxon relative to the number of individuals. For beta diversity, principle coordinates analysis was carried out using the Bray-Curtis similarity index.

### Quantitative PCR for validation

To validate the results of 18S rDNA MiSeq analysis a subset of 36 samples (6% of the total sample set) were selected for quantification of *Eimeria* occurrence and abundance using a validated species-specific quantitative PCR (qPCR) targeting single copy sequence characterized amplified region (SCAR) markers, conducted as published by Vrba and colleagues (10). Subsequently, half (*n* = 18) of these samples were re-tested using a published assay targeting the ITS-2, undertaken as described by Morgan et al. (12). PCR inhibition was screened using VetMAX Xeno Internal Positive Control (Thermo Scientific) and samples were diluted 1:9 with molecular grade water for analysis of 5 μl template in 20 μl reactions. Proportions of each species were calculated using relative quantification (SCAR-based qPCR) or absolute quantification (ITS-2 qPCR). DNA standards with a known amount of DNA representing each species were used in both assays. Results were expressed as a percentage proportion of each of the assayed species or Ct values.

### Statistics

The proportionate abundance of each OTU group was assessed using the non-parametric Kruskal-Wallis test in SPSS (version 24; IBM, New York,USA) as described elsewhere (34, 35).

## Results

### Sequencing results and OTU clustering

In total 19,454,434 reads were obtained from two MiSeq runs sequencing 600 samples and used to generate OTU clusters by comparison with the reference sequence dataset. All sequence data has been deposited at GenBank under the accession numbers SRR7178810-SRR7179409 within Bioproject PRJNA471386. Samples from eight birds which comprised less than 10,000 reads were removed from the study. Rarefaction of the remaining 592 samples approached asymptote and all were included in subsequent analyses (data not shown). OTU clustering identified 118 OTUs in total, using 64.1% of the reads. Genus-level taxonomic assignment identified 99.7% *Eimeria* and 0.3% *Cryptosporidium* sequences within the OTU set, supplemented by a minor *Hyaloklossia lieberkuehni* occurrence (Table 1).

**Table 1:** Average OTU abundance and taxonomy assignment per chicken line/experimental group shown to four significant figures.

**Published taxon ID**	**No. birds positive (% positive)**				**Average representation (%)**				**Kruskal-Wallis**	
	**C**	**K**	**CK_C**	**CK_K**	**C**	**K**	**CK_C**	**CK_K**	**All groups**	**CK_C:CK_K**
*Eimeria tenella*	145 (100)	150 (100)	149 (100)	148 (100)	75.02	69.45	55.81	59.98	0.000	ns
*Eimeria necatrix*	145 (100)	150 (100)	149 (100)	148 (100)	20.10	20.89	33.44	29.53	0.000	ns
^a^unclassified *Eimeria* sp	145 (100)	150 (100)	149 (100)	148 (100)	4.255	4.319	4.377	4.119	ns	ns
^b^*Eimeria cf mitis*	113 (77.9)	106 (70.7)	109 (73.2)	113 (76.4)	0.194	2.907	0.334	0.597	ns	ns
*Eimeria mitis*	83 (57.2)	44 (29.3)	55 (36.9)	56 (37.8)	0.032	2.355	0.271	0.433	ns	0.042
*Eimeria acervulina*	32 (22.1)	15 (10.0)	85 (57.0)	80 (54.1)	0.079	0.002	0.226	0.145	0.001	ns
^c^*Eimeria* sp *Meleagris gallopavo* 226-2	29 (20.0)	27 (18.0)	53 (35.6)	47 (31.8)	0.001	0.002	0.007	0.004	ns	ns
^c^*Eimeria* sp *Alectoris graeca*-29-2-10.s2	20 (13.8)	24 (16.0)	78 (52.3)	79 (53.4)	0.001	0.002	0.023	0.012	ns	ns
*Cryptosporidium meleagridis*	14 (9.6)	28 (18.7)	63 (42.3)	48 (32.4)	0.117	0.046	0.393	0.428	0.003	ns
*Eimeria maxima*	11 (7.6)	13 (8.7)	47 (31.5)	53 (35.8)	0.141	0.024	0.384	0.210	ns	ns
*Eimeria meleagridis*	8 (5.5)	8 (5.3)	14 (9.4)	19 (12.8)	0.000	0.000	0.001	0.001	ns	ns
*Eimeria brunetti*	5 (3.4)	0 (0.0)	101 (67.8)	99 (66.9)	0.021	0.000	3.411	1.810	0.000	ns
*Eimeria praecox*	4 (2.8)	1 (0.7)	118 (79.2)	118 (79.7)	0.033	0.000	1.318	2.715	0.000	ns
^b^*Eimeria* sp *Alectoris graeca*-29-1-10.s1	2 (1.4)	2 (1.3)	12 (8.1)	18 (12.2)	0.000	0.000	0.001	0.001	ns	ns
*Eimeria* OTUz	0 (0.0)	0 (0.0)	6 (4.0)	8 (5.4)	0.000	0.000	0.000	0.001	ns	ns
*Eimeria* OTUy	0 (0.0)	0 (0.0)	4 (2.7)	11 (7.4)	0.000	0.000	0.003	0.001	ns	ns
*Hyaloklossia lieberkuehni*	0 (0.0)	4 (2.7)	9 (6.0)	3 (2.0)	0.000	0.002	0.001	0.003	ns	ns
*Cryptosporidium baileyi*	0 (0.0)	4 (2.7)	0 (0.0)	0 (0.0)	0.000	0.003	0.000	0.000	ns	ns
Total birds for analysis	145	150	149	148						

OTUs represented by less than 0.001% of sequence reads are not shown. C, homogeneous Cobb400 flock; K, homogeneous Kadaknath flock; CK_C, Cobb400 birds sampled from mixed Cobb400/Kadaknath flock; CK_K, Kadaknath birds samples from mixed Cobb400/Kadaknath flock.

a Found by BLASTn to be identical to E. necatrix.

b Sequences attributed to Eimeria mivati.

c *Unclassified. Significant differences in sequence representation determined using the Kruskal-Wallis non-parametric test, applied first to all four groups, and then to compare CK_C and CK_K only*.

**Figure 1:**
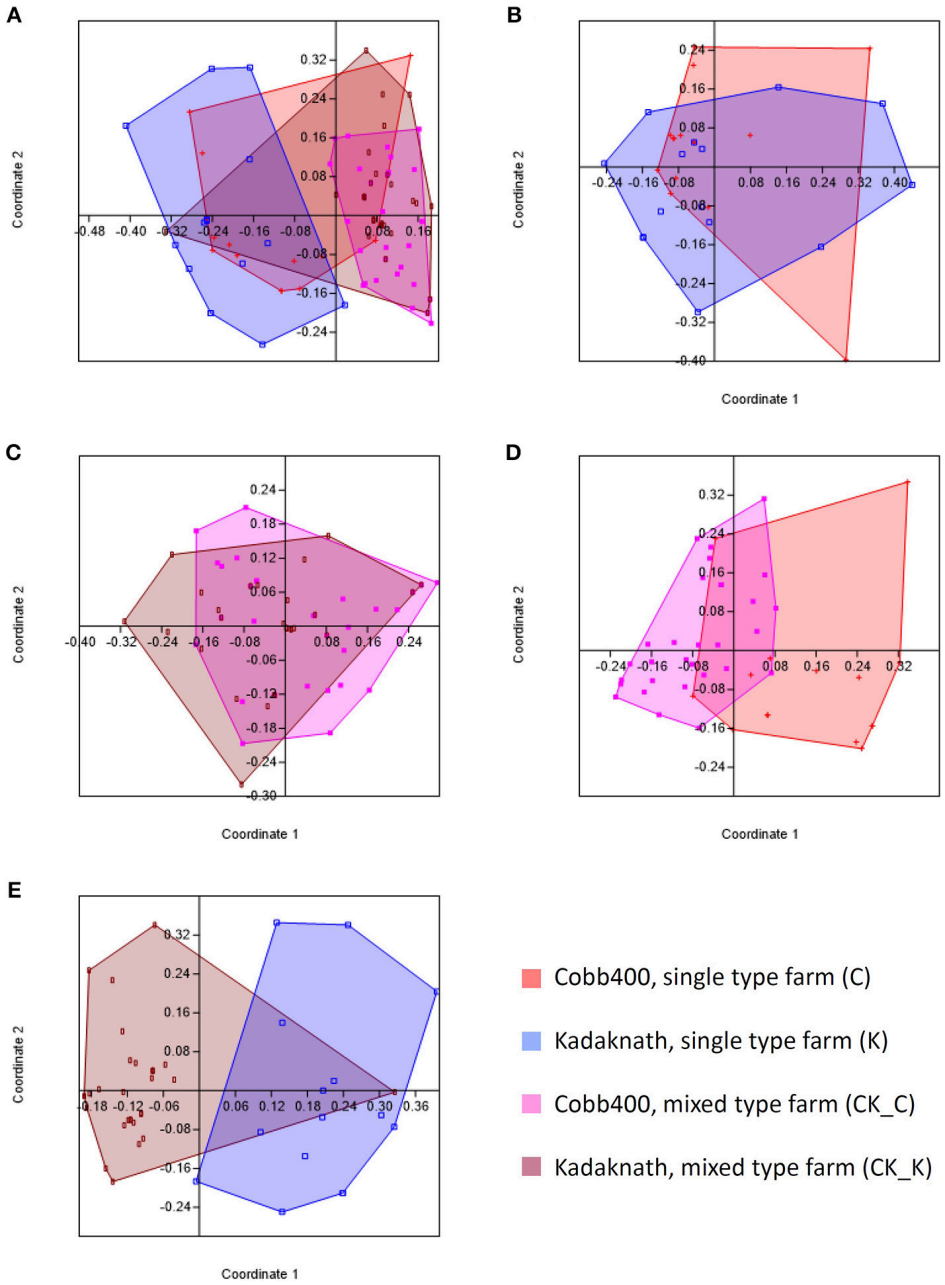
Principle coordinates analyses generated using PAST software where the Bray-Curtis similarity index was used to generate clusters for different chicken populations. **(A)** Comparison of clusters for C, K, CK_C and CK_K datasets. **(B)** Comparison of clusters for C and K datasets. **(C)** Comparison of clusters for CK_C and CK_K datasets. **(D)** Comparison of clusters for C and CK_C datasets. **(E)** Comparison of clusters for K and CK_K datasets.

Comparison of caecal 18S rDNA amplicon-based OTU populations between C, K, CK_C, and CK_K datasets using PCA revealed different profiles for all (Figure 1A). Pairwise comparison revealed equivalent levels of similarity between Cobb400 and Kadaknath when reared individually on single line farms or together in mixed farm flocks (Figures 1B,C), and for Cobb400 reared in single line or mixed flocks (Figure 1D). The greatest variation was noted between Kadaknath reared in single line and mixed flocks (Figure 1E).

### Occurrence of parasite species

Analysis of sequence datasets from 592 individual chickens revealed the presence of DNA representing one or more *Eimeria* species in every sample. Specifically, every sample included *E. tenella* and *E. necatrix* 18S rDNA, consistent with the use of caecal contents for template preparation (Table 1). Additionally, low levels of DNA with greatest similarity to accession number KU160243 (unclassified *Eimeria* species, recovered from Northern bobwhite quail) were also detected in every sample. *Cryptosporidium meleagridis* was the most commonly detected non-eimerian, supplemented by *Cryptosporidium baileyi* in a small number of Kadaknath only flocks (Table 1). DNA representing the parasitic alveolate *Hyaloklossia lieberkuehni* was detected on a small number of farms in all groups except the Cobb400 only flocks (Table 1).

### Taxonomic composition

Consideration of the abundance of *Eimeria* species within all four datasets found *E. tenella* to be dominant (8,079,670 reads in total), followed by *E. necatrix* (3,244,923)*. Eimeria tenella* was detected in all groups, on average representing between 56 and 75% of reads from Cobb400 birds reared in mixed and single breed flocks respectively, and 60 and 70% of Kadaknath from mixed and single breed flocks (Table 1; those with >10,000 reads per sample). Significant differences in sequence abundance were detected between single and mixed breed flocks (*p* < 0.001), but not between chicken breeds. *Eimeria necatrix* represented 20 to 33% of sequences, being highest in the mixed flock Cobb400 and Kadaknath (*p* < 0.001), but again not different between breeds. *Eimeria mitis* (223,166 total reads), *E. brunetti* (164,649), *E. praecox* (130,601), *E. maxima* (23,585), and *E. acervulina* (14,096) were also detected, as was *E. meleagridis* at a very low level (75), a parasite classically associated with turkeys. Two of the three cryptic *Eimeria* genotypes first described in Australia were detected at very low abundance [OTUy (127) and OTUz (56), but not OTUx; (3)]. A notable proportion of sequences were most closely related to an as yet unclassified *Eimeria* sequence derived previously from a Northern bobwhite quail (*Colinus virginianus*; 530,804), although subsequent BLASTn comparison found this sequence to be identical to *E. necatrix*. A small number of sequences from *Eimeria* of wild turkey (*Meleagris gallopavo*) and rock partridge (*Alectoris graeca*) were also detected, possibly illustrating farm-level contamination with non-replicating *Eimeria* oocysts or DNA. Comparison of the percentage occurrence of each *Eimeria* species between Cobb400 and Kadaknath reared in mixed flocks (experimental group 1) revealed a significant difference only for *E. mitis*, appearing at a significantly higher abundance in Kadaknath chickens (Table 1; *p* < 0.05), reflecting a larger difference between Cobb400 and Kadaknath reared in single breed flocks. In agreement with the farm-level occurrence, *C. meleagridis* was the most abundant non-eimerian, supplemented by *C. baileyi* and *H. lieberkuehni* (Table 1). Assessment of the less abundant taxa revealed the influence of varied occurrence in a small number of flocks. For example, the individually reared Cobb400 showed a comparatively higher abundance of *C. meleagridis* due to an above average representation on a single farm (C-09).

### Comparison of NGS data with quantitative PCR

In order to validate the 18S rDNA NGS protocol a subset of 36 caecal gDNA samples were selected for quantitative detection of *Eimeria* species known to infect chickens using qPCR targeting single copy species-specific SCAR markers. Such quantitative data can also be used to benchmark NGS data, permitting sequence read numbers to be used as semi-quantitative data. Between one and seven *Eimeria* species were detected in all 36 samples using MiSeq (Supplementary Table 3). No *Eimeria* were detected in 20 of 36 samples by SCAR qPCR. Between two and seven *Eimeria* species were detected by SCAR qPCR in the remaining 16 samples, 13 of which identified the same dominant *Eimeria* species detected by MiSeq. There was one example of an *Eimeria* species detected by SCAR qPCR but not MiSeq (*E. mitis*; Supplementary Table 3).

Subsequently, considering the low level of detection achieved by the SCAR qPCR a subset of 18 of the same samples were further subjected to species-specific qPCR targeting the multi-copy ITS-2 locus. Comparison of MiSeq data with ITS-2 qPCR revealed a limit of detection comparable to SCAR qPCR, with between one and three *Eimeria* species detected in 7/18 samples. However, consistency was also low between the SCAR and ITS-2 qPCR since just 4/18 samples were found to contain the same *Eimeria* species by both assays. The considerable differences reported between MiSeq NGS and qPCR might reflect differences in sensitivity.

### Diversity analysis

The taxonomic data generated was used to estimate a range of alpha diversity indices (Table 2). When Cobb400 and Kadaknath birds were reared separately, the abundance of species related to *Eimeria* revealed approximately 8 species per individual in both types of chicken (Taxa_S), rising to 11 when Cobb400 and Kadaknath were reared together. The Dominance_D index was lowest in Kadaknath and Cobb400 when reared together. The high Simpson's index values indicate an even distribution of different taxa within either breed from mixed flocks compared to those reared in single breed flocks. Furthermore, like the Dominance_D and Simpson's indices, the other alpha diversity indices also revealed the highest microbial diversity for Cobb400 and Kadaknath from mixed flocks (Table 2).

**Table 2:** Summary of diversity indices.

	**C**	**K**	**CK_C**	**CK_K**
Taxa_S	9	9	13	12
Dominance	0.614	0.558	0.512	0.530
Simpson	0.386	0.442	0.488	0.470
Shannon	0.690	0.783	0.866	0.823
Berger-Parker	0.754	0.698	0.645	0.671
Chao-1	9.133	8.933	12.567	11.633

C, homogeneous Cobb400 flock; K, homogeneous Kadaknath flock; CK_C, Cobb400 birds sampled from mixed Cobb400/Kadaknath flock; CK_K, Kadaknath birds samples from mixed Cobb400/Kadaknath flock

## Discussion

Molecular assays for the detection and quantification of *Eimeria* in chickens have been available for many years, although techniques based upon microscopy and intestinal pathology are still more widely used (36). While molecular tools can improve accessibility to species-specific *Eimeria* diagnostics, the occurrence of cryptic *Eimeria* genotypes may prove limiting to sequence-specific assays such as PCR (3). In this study the occurrence of *Eimeria* parasites and closely related organisms within the chicken caecal lumen has been assessed using a new NGS assay targeting the 18S rDNA. NGS has been used previously to explore the occurrence of eimerian parasites in brush-tailed rock-wallabies (18), and it is now timely to develop the approach further for use with *Eimeria* which infect livestock. Key areas of interest include levels of naturally occurring parasite population diversity, and assessment of the impact of treatments such as chemoprophylaxis, vaccination, dietary modulation or probiotics. It is important to note that the samples used here were collected for use in a bacterial microbiome study, and as such additional samples from other sites within the intestine or faecal material were not available. Analysis of multiple intestinal sites and/or faecal material would provide a valuable comparison and a more global assessment. Indeed, the work described was not intended to create a comprehensive analysis of *Eimeria* occurrence in each chicken, rather to explore the use of 18S rDNA deep amplicon sequencing to define specific eimerian populations and inform upon potential parasite-bacterial interactions.

Analysis of 592 individual chicken caecal samples revealed the presence of eimerian DNA in every example, including at least two species in each. The detection of eimerian DNA does not definitively prove that all chickens were infected, since dietary or environmental contamination with *Eimeria* nucleic acids cannot be ruled out. However, such a high level of occurrence does indicate a wide distribution for these parasites. A similarly high level of occurrence has been described previously in chickens sampled from South East India (Tamil Nadu) using qPCR (36). *Eimeria tenella* was most common, appearing as the dominant OTU in most individuals. *Eimeria tenella* specifically invades and replicates in caecal epithelial cells (37) so it is unsurprising to see such a high representation given the use of caecal lumen contents as template for this study. *Eimeria necatrix*, a parasite which undergoes the sexual stages of it lifecycle in the caeca (5), was the second most abundant OTU. The third most prevalent OTU detected was associated with an unclassified *Eimeria* sequence derived previously from a Northern bobwhite quail, although the sequence was subsequently annotated as *E. necatrix* by BLASTn sequence similarity. This finding may illustrate a genuine lack of sequence diversity between *Eimeria* species, or perhaps more likely highlight a difficulty associated with the use of public sequence resources where errors in sequence annotation can yield misleading results (38). While the breadth provided by accessing all publically available data is a considerable strength, it is clear that stringent biological interpretation is required. The use of a smaller, manually curated reference dataset might be expected to reduce the risk of such mis-annotation, although some important sequences might remain unannotated as a consequence. In the future the use of targets such as mtCOI could offer valuable alternatives (21), although accurate reference sequence annotation will remain essential.

*Eimeria* species such as *E. maxima, E. acervulina, E. praecox* and *E. brunetti* were also detected at low levels in the caecal lumen 18S rDNA dataset. While these parasites are unlikely to have been replicating in the caeca (5), it is likely that parasites (and parasite DNA) produced by infections higher up the gastrointestinal tract may have been transiting through the caeca at the time of sampling. Two of the three cryptic *Eimeria* genotypes were detected at very low levels within the NGS dataset, including OTUy for the first time in India (3), providing the most northern recording of this genotype to date. No difference was detected in occurrence of these two genotypes between commercial and indigenous chicken breeds, prompting the hypothesis that their polarised global distribution is unlikely to be an artefact of host genotype specificity.

Differences in *Eimeria* species occurrence were more frequently detected between chickens of the same breed reared in single and mixed breed flocks than between the two chicken breeds, indicating a stronger role for environment and management than host genetic effects. It is possible that the process of introducing chickens from different backgrounds into mixed farm systems may have increased *Eimeria* occurrence and population complexity (3). Only *E. mitis* occurrence was found to be significantly different between Cobb400 and Kadaknath chickens reared in shared flocks, a finding which was replicated in the comparison of these two breeds from single breed flocks. *Eimeria mitis* presents an intermediate level of risk to chicken health (39) and the identification of resistant individuals is likely to be of value to future breeding strategies. High levels of variation associated with the hybrid background of the commercial Cobb400, and possible local variation in indigenous Kadaknath populations, may have obscured variation in occurrence for other *Eimeria* species. The age at which chickens were sampled may also have contributed to the lack of heterogeneity, since it is likely that many would have experienced prior infections, indicating the presence of an ongoing partially protective immune response. The balance between parasite re-infection and protective immunity may have resulted in a situation of enzootic stability as has been described for other apicomplexan parasites (40), smoothing out breed-specific variation and limiting parasite abundance.

Two quantitative PCR protocols were applied to subsets of the samples defined by 18S rDNA NGS to provide biological positive control. In all but one example the identity of the dominant *Eimeria* species was validated by at least one of the qPCR assays. Comparison of results for the minority OTUs suggested significantly greater sensitivity for the 18S rDNA NGS, likely permitting detection of genomic DNA originating from *Eimeria* species replicating higher up the gastrointestinal tract and transiting through the caeca. As an alternative, it is possible that even residual non-replicating parasite material ingested from the environment might have been detected. Such apparent variation in sensitivity remains a pertinent question with a high false-positive rate an alternative explanation. Future development of NGS approaches for use with *Eimeria* would benefit from the inclusion of additional control samples such as defined mixtures of pure eimerian genomic DNA. Knowledge defining *Eimeria* species occurrence can be of value to flock productivity and welfare, but beyond validation of the NGS protocol quantitative abundance is required to determine the risk of disease. Similarly, parasite presence/absence is not necessarily a measure of host resistance to a parasite. Levels of colonisation and replication are more relevant. Quantitative PCR can be used to benchmark NGS microbiome data and facilitate a direct assessment of parasite load, and thus risk of disease. However, the lower sensitivity of the qPCR protocols for OTUs represented by low read coverage in individual samples precluded such analysis here. It is important to note that the detection of *Eimeria*, or eimerian DNA, does not inevitably associate with clinical disease, although it should encourage improved anticoccidial control through husbandry, chemoprophylaxis and/or vaccination.

The detection of *E. meleagridis* and unclassified OTUs associated with other *Eimeria* of *M. gallopavo* and *A. graeca* is intriguing. Given the high sensitivity of the 18S rDNA NGS protocol the low levels detected may merely represent dietary or environmental contamination with non-replicating oocysts or parasite DNA. The number of reads representing each of these species were very low and could have represented low level experimental contamination, although the absence of such reads in the negative controls and their limited distribution through the sample panel suggest that this was unlikely. It has been noted that *E. meleagridis* is capable of replicating in alternative hosts such as grey partridge (*Perdix perdix*) in addition to the turkey, although no evidence of replication has been detected in chickens (41). Similarly, the detection of *C. meleagridis* may indicate low levels of replication by this zoonotic parasite (42) and a possible health risk to consumers of poultry products, or farm-level contamination with faecal material from other poultry.

The work described here provides the first example of NGS deep 18S rDNA amplicon analysis to define *Eimeria* parasite populations in chickens. Application of the protocol has suggested notable sensitivity compared to two well established qPCR assays. Comparison of *Eimeria* occurrence between commercial and indigenous chickens bred and reared in India revealed significant variation only for *E. mitis*.

## Author contributions

AH did data analysis and initial manuscript writing. JT provided sample processing, DNA isolation and sequencing of samples. PK and SJ did sample collection and manuscript correction. VV contributed data production and manuscript writing. AP and JG designed experiments and reviewed the manuscript. CJ, DR, MR, FT and DB were involved in acquisition of funding, experimental design, and review of the manuscript.

### Conflict of interest statement

The authors declare that the research was conducted in the absence of any commercial or financial relationships that could be construed as a potential conflict of interest.
